# Supplementary material for: Quantification of frequency-dependent genetic architectures in 25 UK Biobank traits reveals action of negative selection
Source: Nat Commun. 2019 Feb 15;10:790. doi: 10.1038/s41467-019-08424-6 (PMC6377669; doi:10.1038/s41467-019-08424-6)
Supplement: Supplementary file 1 — Supplementary Information [file 41467_2019_8424_MOESM1_ESM.pdf]

Supplementary Information for "Quantification of frequency-dependent genetic architectures in 25 UK Biobank traits reveals action of negative selection"

Schoech et al.

Supplementary Figures:

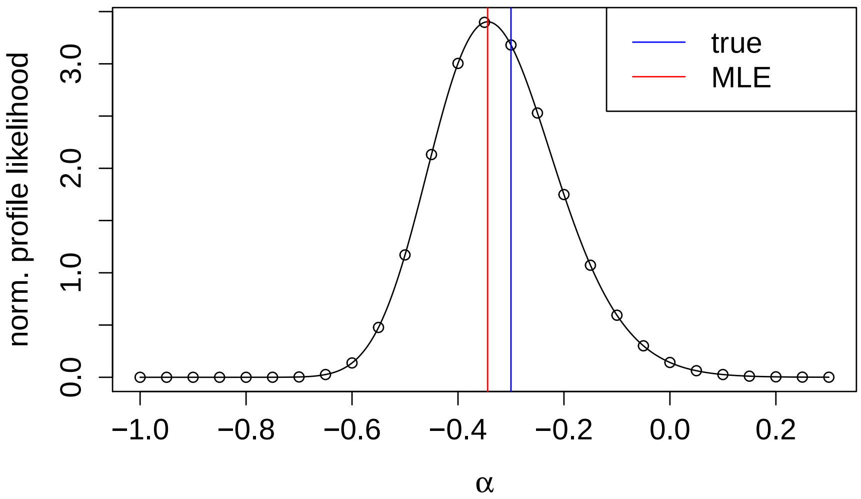

Supplementary Figure 1: Profile likelihood curve from a random sample simulation with $\alpha = -0.3$ and $h_g^2 = 0.4$. The MLE $\hat{\alpha} = -0.34$. The curve is smooth and unimodal, as it is the case for all simulations. Profile likelihood points calculated with GCTA are interpolated by a standard cubic spline and the likelihood values were normalized such that the total area under the curve is 1.

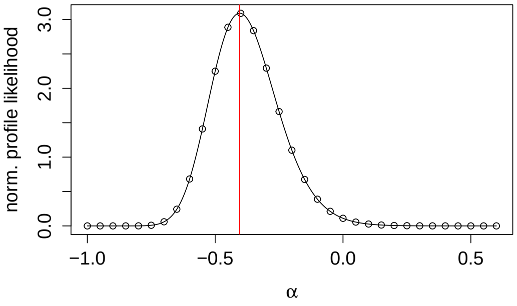

(a) age of menarche

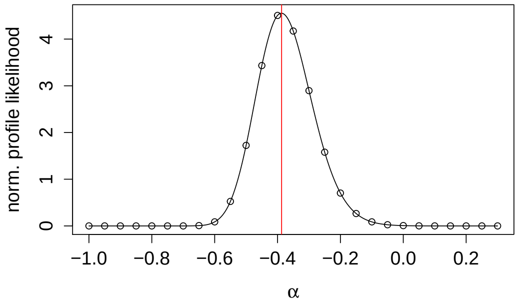

(b) blood pressure (diastolic)

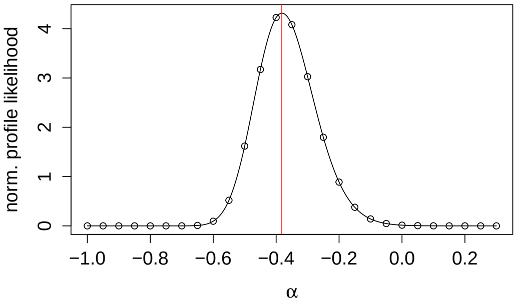

(c) blood pressure (systolic)

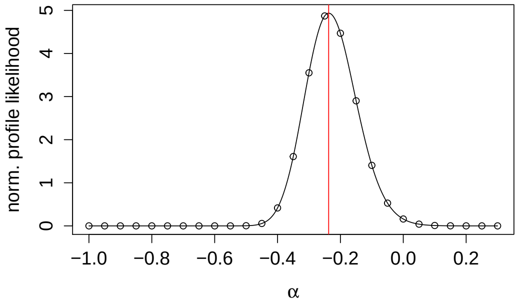

(d) BMI

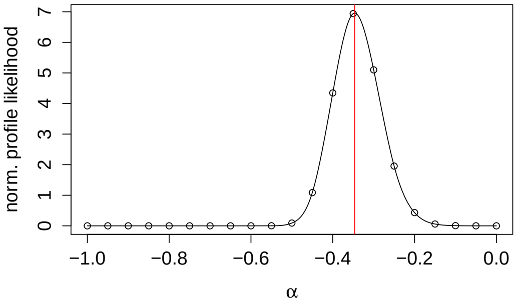

(e)
bone mineral density

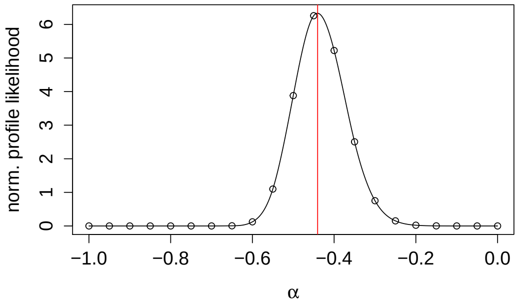

(f) FEV1/FVC

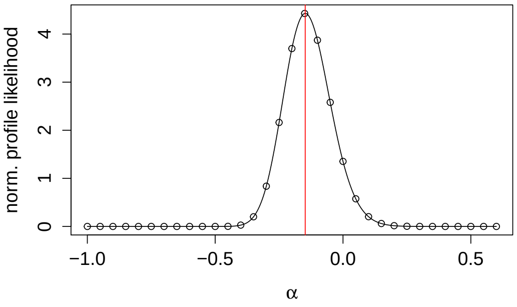

(g) FVC

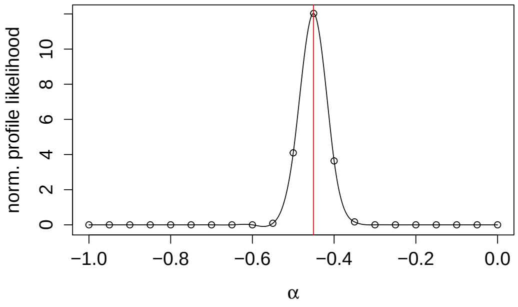

(h) height

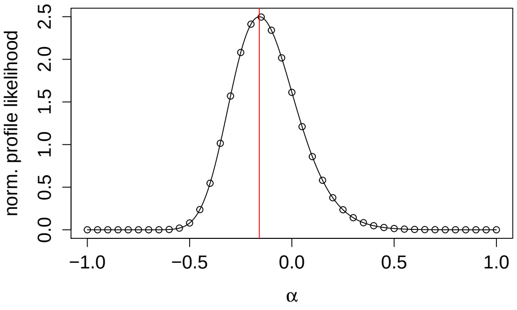

(i) smoking status

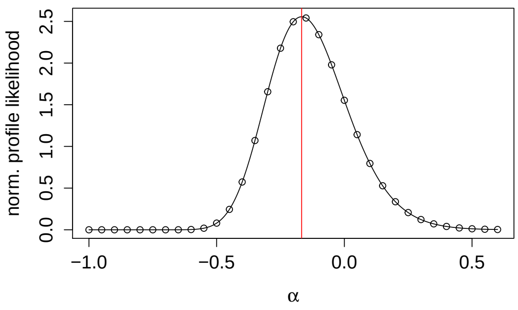

(j) waist-hip ratio

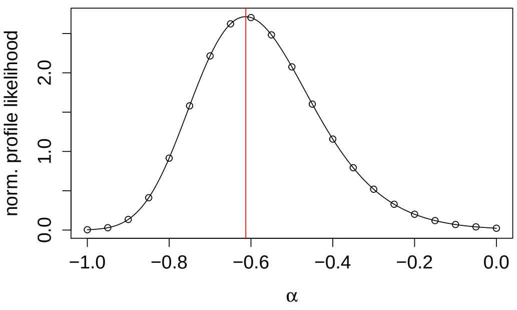

(k) allergic eczema

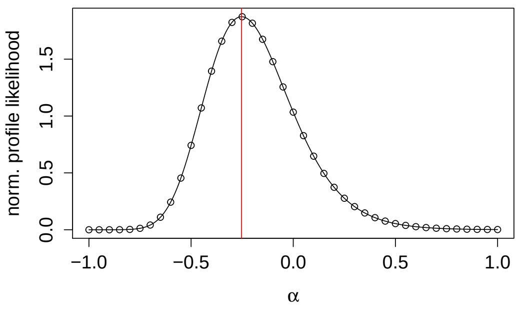

(l) asthma

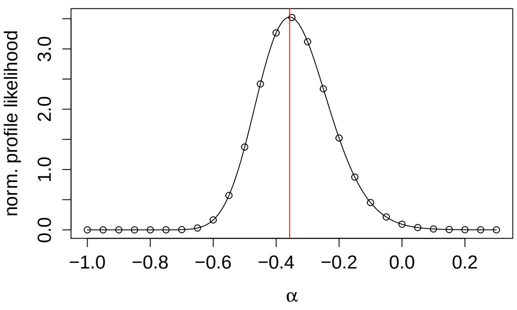

(m) college education

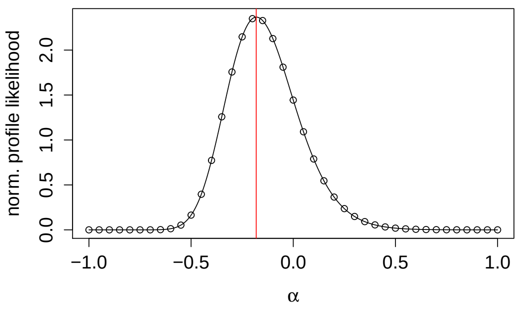

(n) hypertension

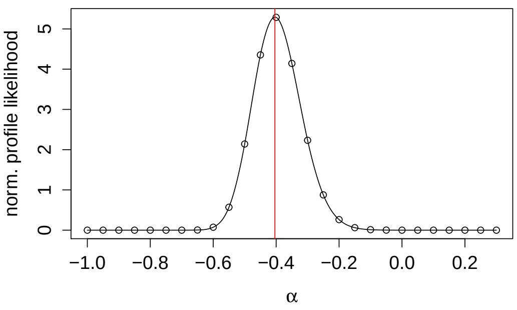

(o) eosinophil count

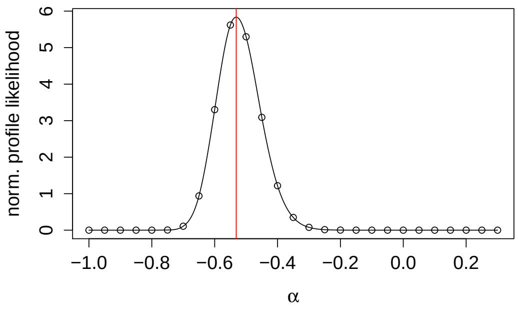

(p) high light scatter reticulocyte count

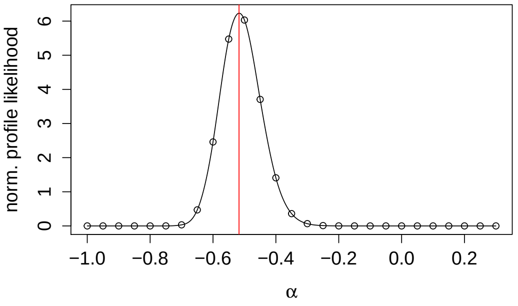

(q) lymphocyte count

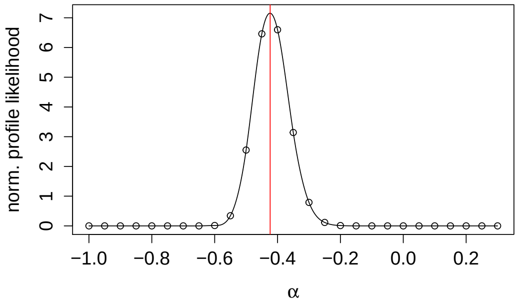

(r) mean corpuscular hemoglobin

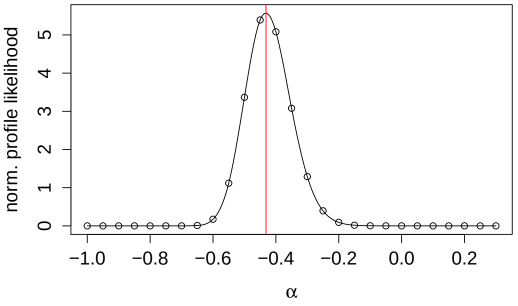

(s) mean sphered cell volume

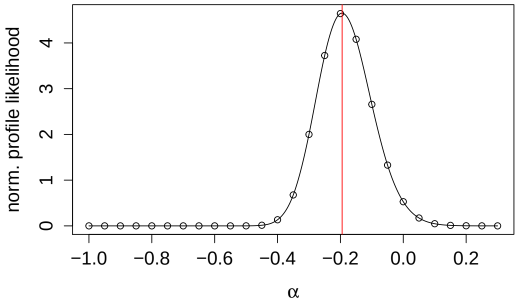

(t) monocyte count

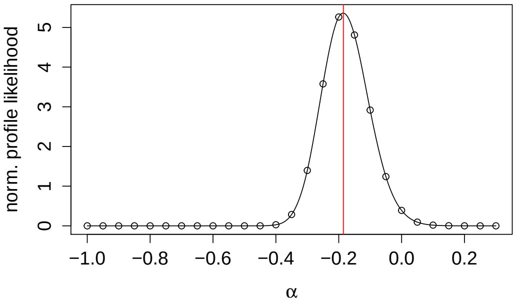

(u) platelet count

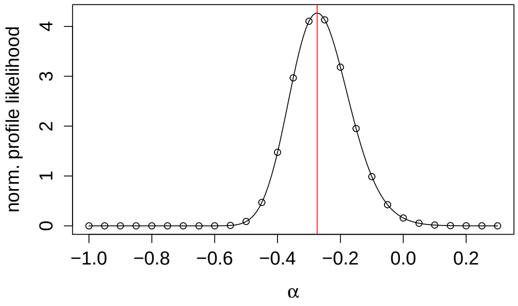

(v) platelet distribution width

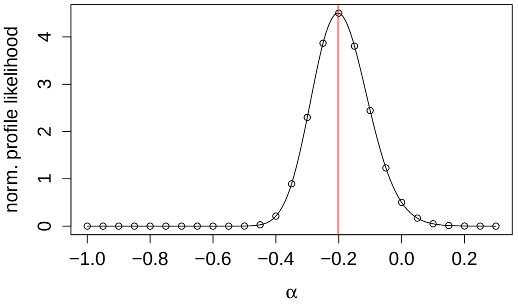

(w) red blood cell count

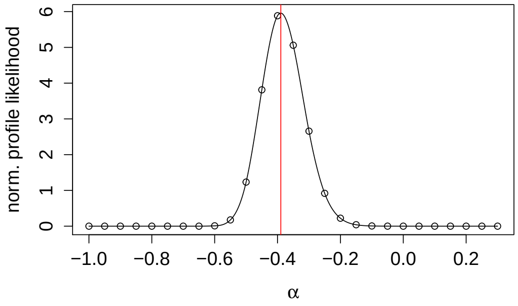

(x) red blood cell distribution width

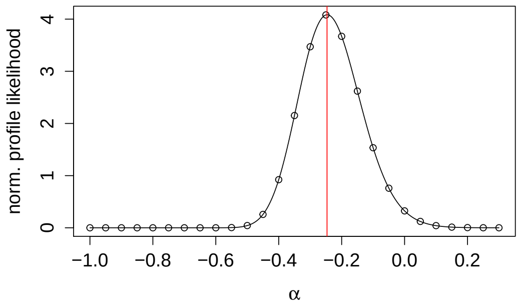

(y) white blood cell count

Supplementary Figure 2: Profile likelihood curve calculated for 25 UK Biobank traits analyzed. Data points were interpolated using a natural cubic spline and the likelihood values were normalized such that the total area under the curve is 1. The red vertical line at the mode of the curve represents the estimate of $\hat{\alpha}$. Numerical values of $\hat{\alpha}$ are reported in Table 2.

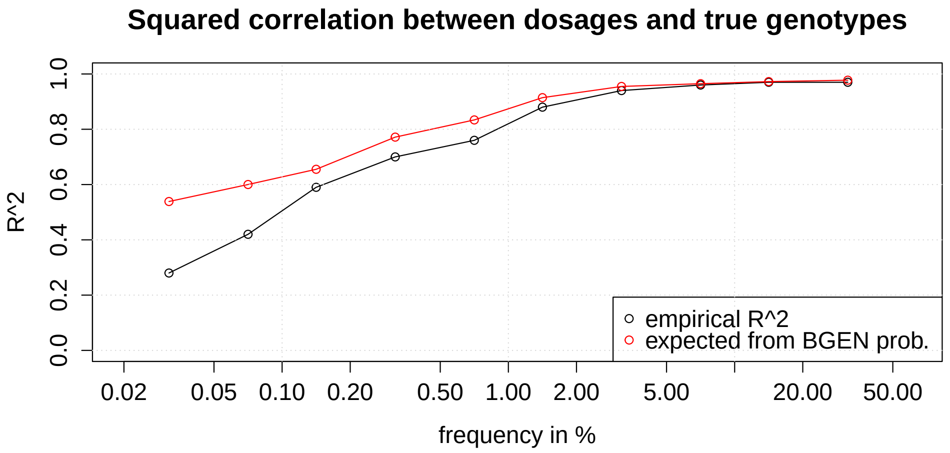

plot.pdf

Supplementary Figure 3: Comparing empirical UK Biobank imputation accuracy (UK Biobank documentation:
http://www.ukbiobank.ac.uk/wp-content/uploads/2014/04/imputation_documentation_May2015.pdf) with accuracy implied by genotype imputation probabilities from BGEN files for different minor allele frequency ranges. To calculate the implied accuracy, 10 British UK Biobank individuals where taken at random and the average imputation R^2 implied by the genotype probabilities was calculated for SNPs of a given frequency range. Imputation accuracy is strongly overestimated for SNPs with MAF $\lesssim 0.1\%$, very well calibrated for SNPs with MAF $\gtrsim 1\%$, and slightly overestimated in between.

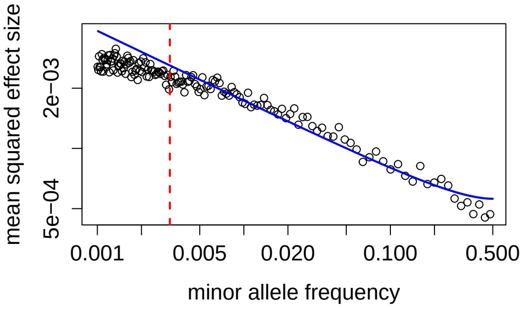

(a) $\bar{s} = 10^{-3}, k = 0.125, \tau = 0.4$

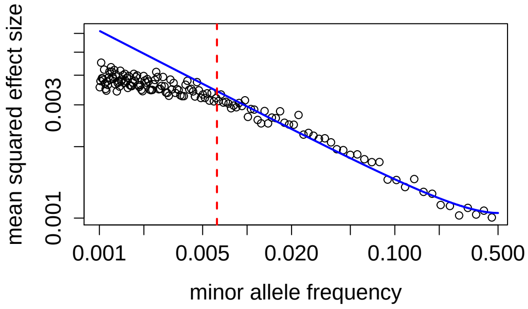

(b) $\bar{s} = 10^{-3}, k = 0.25, \tau = 0.4$

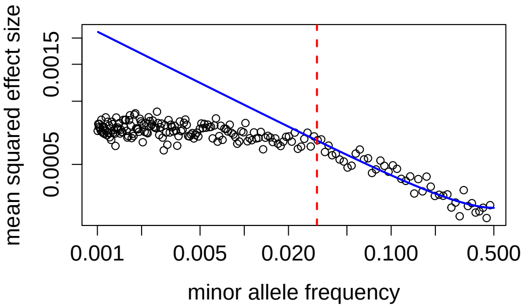

(c) $\bar{s} = 10^{-4}, k = 0.125, \tau = 0.4$

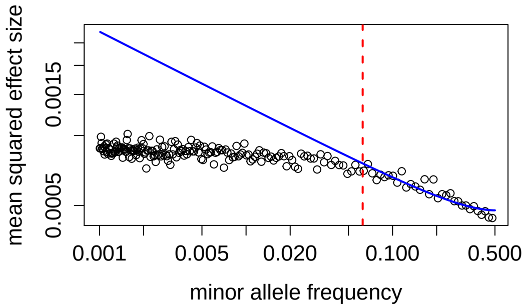

(d) $\bar{s} = 10^{-4}, k = 0.25, \tau = 0.4$

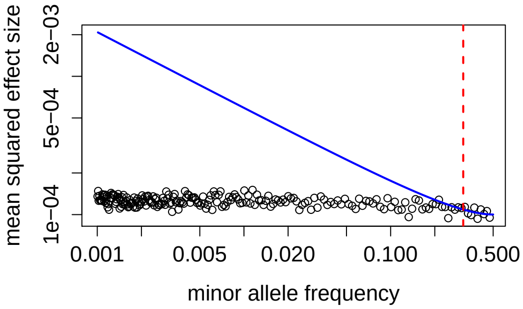

(e) $\bar{s} = 10^{-5}, k = 0.125, \tau = 0.4$

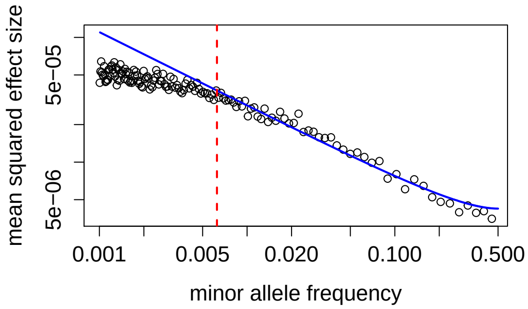

(f) $\bar{s} = 10^{-3}, k = 0.25, \tau = 0.8$

Supplementary Figure 4: Simulated mean squared SNP effect sizes at a given MAF on a log-log plot. Forward simulations using the SLiM2[8] software were performed using distribution of fitness effects for *de novo* mutations with different average $\bar{s}$, gamma shape parameter k and τ (see subcaptions). Data points represent the mean squared effect size of 1000 SNPs of similar MAF. The blue line represents the mean squared effect size under the α model (see equation 1), when α is fitted to SNPs above the MAF threshold of $T = \frac{k}{4N_4\bar{s}}$, which indicates the breakdown of the model for rarer SNPs (dashed red line). We did not test $\bar{s} \geq 10^{-2}$ since this would not be plausible for genome wide SNPs. Furthermore, we do not display simulations with $\bar{s} < 10^{-5}$ since in this case $T \geq 50\%$ for plausible k, and hence the α model cannot be fitted. Values of k tested include the plausible range reported by[9].

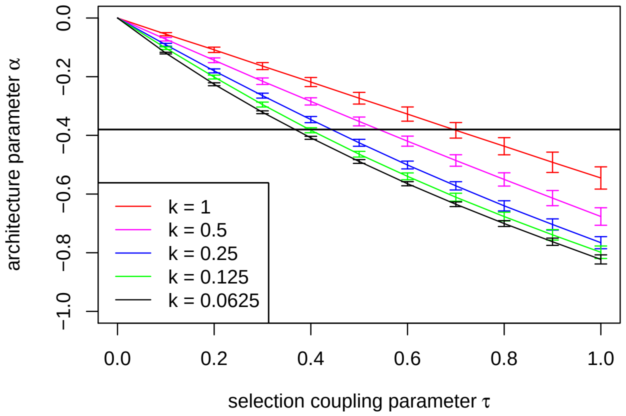

(a) Average genome wide selection coefficient $\bar{s} = 10^{-3}$.

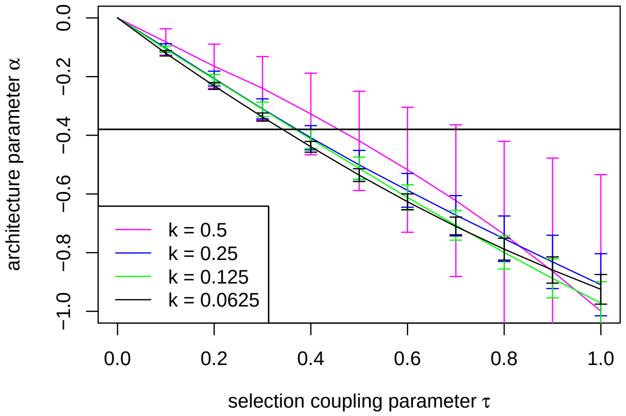

(b) Average genome wide selection coefficient $\bar{s} = 10^{-4}$

Supplementary Figure 5: α-τ mapping using a wider range of SNP fitness effect distributions. We report best-fit estimates of α when varying the selection coupling parameter τ (Eyre-Walker 2010 PNAS) in forward simulations (see Online Methods). In these simulations, negative selection coefficients of SNPs were randomly sampled from a gamma distribution with varying shape parameter k and mean 10^{-3} (a) or 10^{-4} (b). This plot is similar to Figure 3, only with a wider range of k values considered. Bootstrap standard errors across 25 independent simulations are shown. The horizontal dashed line indicates $\alpha = -0.38$, the best-fit α across the 25 UK Biobank traits. Only SNPs above twice the MAF threshold ($2T = \frac{k}{2N_e\bar{s}}$) were used when fitting α, in order to avoid edge effects near the threshold. A curve with $\bar{s} = 10^{-4}$ and $k = 1$ is not shown because this would correspond to $2T = 0.5$ with no SNPs left to fit α. The $\bar{s} = 10^{-4}$ and $k = 0.5$ curve has very large error bars, because there are only few SNPs with MAF $> 2T = 0.25$ in these simulations to be used when fitting α. Both $\bar{s} = 10^{-4}$ with $k = 1$ and $\bar{s} = 10^{-4}$ with $k = 0.5$ are very unlikely since these would correspond to a threshold T of 25% and 12.5% respectively, with no frequency dependence for SNPs of MAF below T. As we argue in the Results section, T is likely lower than 5%. We hence do not include these two parameter settings when calculating the range of possible τ values given the best-fit α value from the analyzed UK Biobank traits.

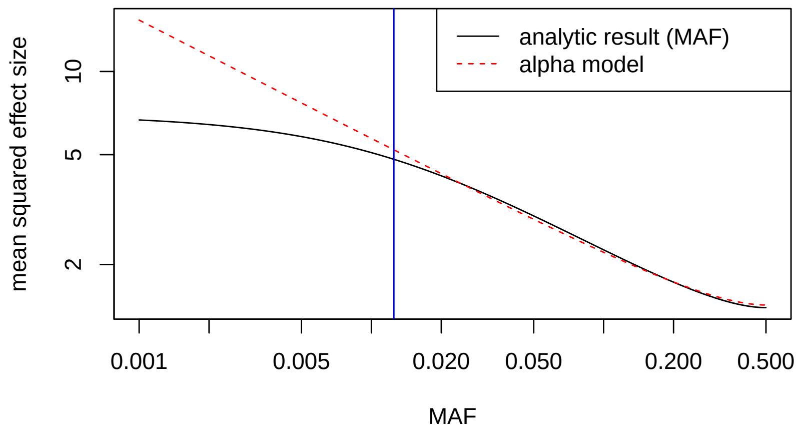

(a)

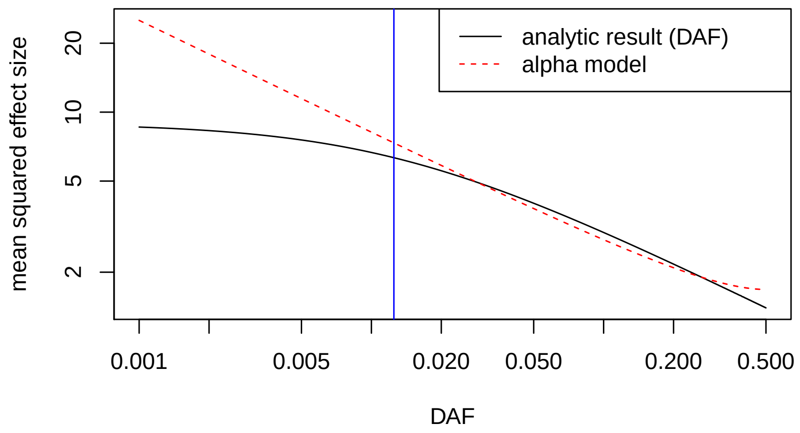

(b)

Supplementary Figure 6: MAF and DAF dependent analytic results compared to the α model. In (a), the MAF-dependent architecture implied by an analytic evolutionary model is compared to the α model (see equation 1). We assume no LD between effected sites and a constant effective population size $N_e = 10,000$, as well as a gamma distributed DFE with mean $\bar{s} = 10^{-3}$ and shape $k = 0.25$. Under these assumptions $P(p|s) \propto \frac{1}{1+e^{-4N_e s}} \cdot [p(1-p)]^{-1}[e^{-4N_e sp} + e^{-4N_e s(1-p)}]$ for a given SNP with selection coefficient s and MAF p (Ewens, WJ. *Mathematical Population Genetics*. Springer, 2004). It follows that: $\mathrm{E}(\beta^2|p) = c \cdot \mathrm{E}(s^{2\tau}|p) = c \cdot \frac{\int_0^\infty s^{2\tau} P(p|s)P(s)ds}{\int_0^\infty P(p|s)P(s)ds} \approx c \cdot \frac{\Gamma(2\tau+k)}{\Gamma(k)}(4N_e)^{-2\tau} \frac{[p+T]^{-2\tau-k}+[(1-p)+T]^{-2\tau-k}}{[p+T]^{-k}+[(1-p)+T]^{-k}}$, with threshold frequency $T = \frac{k}{4N_e\bar{s}}$. As we show in (b), this is similar to the simpler result previously derived when p is the derived frequency (see Online Methods), and only significantly differs from it for very common SNPs, where the MAF-based model fits the α model more closely. The fitted α model is depicted using the dashed red line, using only data points above the threshold T (blue line) to fit the α model. In this case $\tau = 0.25$ and the fitted $\hat{\alpha} = -0.43$, close to the previously derived no LD approximation of $\alpha = -2\tau$ (see Online Methods).

Supplementary Tables:

Supplementary Table 1: Assessing calibration of error estimates and bias of $\hat{\alpha}$ in simulations

(a) Comparison of empirical and estimated error in $\hat{\alpha}$ using $N = 5,000; M = 100,000$

α	h_g^2	poly-genicity	imput. noise	LD dep. effects	empirical error	mean error estimate
-0.3	0.4	1%	yes	yes	0.174 ± 0.014	0.139
0	0.4	1%	yes	yes	0.196 ± 0.011	0.136
-0.6	0.4	1%	yes	yes	0.138 ± 0.013	0.109
-0.3	0.2	1%	yes	yes	0.243 ± 0.016	0.205
-0.3	0.4	100%	yes	yes	0.120 ± 0.008	0.141
-0.3	0.4	1%	no	yes	0.163 ± 0.014	0.139
-0.3	0.4	1%	yes	no	0.169 ± 0.020	0.123

(b) Comparison of empirical and estimated error in $\hat{\alpha}$ using $N = 15,000; M = 860,000$

α	h_g^2	poly-genicity	imput. noise	LD dep. effects	empirical error	mean error estimate
-0.3	0.4	1%	yes	yes	0.099 ± 0.008	0.091
0	0.4	1%	yes	yes	0.120 ± 0.009	0.104
-0.6	0.4	1%	yes	yes	0.072 ± 0.006	0.068
-0.3	0.2	1%	yes	yes	0.173 ± 0.015	0.157
-0.3	0.4	100%	yes	yes	0.085 ± 0.006	0.089
-0.3	0.4	1%	no	yes	0.090 ± 0.005	0.088
-0.3	0.4	1%	yes	no	0.082 ± 0.005	0.078

(c) Bias in α estimates using $N = 15,000; M = 860,000$

α	h_g^2	poly-genicity	imput. noise	LD dep. effects	mean $\hat{\alpha}$
-0.3	0.4	1%	yes	yes	-0.288 ± 0.010
0.0	0.4	1%	yes	yes	0.009 ± 0.012
-0.6	0.4	1%	yes	yes	-0.588 ± 0.007
-0.3	0.2	1%	yes	yes	-0.312 ± 0.009
-0.3	0.4	100%	yes	yes	-0.291 ± 0.009
-0.3	0.4	1%	no	yes	-0.291 ± 0.009
-0.3	0.4	1%	yes	no	-0.371 ± 0.008

In (a) and (b), we show the empirical standard deviation of $\hat{\alpha}$ from 100 simulations and the average estimated standard error (see Online Methods) for each genetic architecture tested in Table 1. The empirical standard deviation is reported together with its standard error. In (a), we used a sample size of 5,000 and 100,000 SNPs (the same as in Table 1), while in (b) the sample size was 15,000 and 860,000 SNPs (approximate number of imputed SNPs on chromosome 1 in UK Biobank). Error estimates are slightly lower than the empirical error in (a), but are not significantly biased in (b). In (c), we replicate results shown in Table 1, only now using a larger sample size and SNP number (15,000 and 860,000 respectively), with results being not significantly different from Table 1.

Supplementary Table 2: Heritability estimation from simulated phenotypes with and without MAF and LD correction

α	h_g^2	poly-genicity	imput. noise	LD dep. effects	$\hat{h}^2_{\text{GCTA}}$	$\hat{h}^2_{\alpha,\text{noLD}}$	$\hat{h}^2_{\alpha}$
-0.3	0.4	1%	yes	yes	0.470 ± 0.002	0.395 ± 0.002	0.397 ± 0.002
0	0.4	1%	yes	yes	0.475 ± 0.002	0.398 ± 0.002	0.395 ± 0.002
-0.6	0.4	1%	yes	yes	0.445 ± 0.002	0.383 ± 0.002	0.394 ± 0.002
-0.3	0.2	1%	yes	yes	0.250 ± 0.002	0.196 ± 0.002	0.202 ± 0.002
-0.3	0.4	100%	yes	yes	0.464 ± 0.002	0.390 ± 0.002	0.393 ± 0.002
-0.3	0.4	1%	no	yes	0.464 ± 0.002	0.390 ± 0.002	0.393 ± 0.002
-0.3	0.4	1%	yes	no	0.468 ± 0.002	0.396 ± 0.002	0.401 ± 0.002

Using simulated trait values from 10,000 UK Biobank individuals (see Online Methods), we tested our heritability estimation method against GCTA single variance component REML[1]. The GCTA estimate $\hat{h}^2_{\text{GCTA}}$ overestimates h_g^2 under all genetic architectures tested. Our MAF-corrected estimate $\hat{h}^2_{\alpha,\text{noLD}}$ as well as our MAF and LD-corrected estimate $\hat{h}^2_{\alpha}$ are approximately unbiased. Other methods of avoiding bias due to MAF-dependent and LD-dependent architectures have recently been proposed, including GREML-LDMS[2] and LDAK[3]; a complete benchmarking of SNP-heritability estimation methods is provided elsewhere[4]

Supplementary Table 3: Estimates of α without LD correction for 25 UK Biobank traits using different MAF cutoff for analyzed SNPs

phenotype	$\hat{\alpha}_{\mathrm{noLD}}$ [95% CI]	$\hat{\alpha}_{\mathrm{noLD,MAF>0.3\%}}$ [95% CI]
age of menarche	-0.24 [-0.49, 0.08]	-0.23 [-0.47, 0.09]
blood pressure (diastolic)	-0.28 [-0.45, -0.07]	-0.29 [-0.45, -0.07]
blood pressure (systolic)	-0.28 [-0.46, -0.06]	-0.28 [-0.46, -0.06]
BMI	-0.11 [-0.26, 0.07]	-0.10 [-0.26, 0.08]
bone mineral density	-0.23 [-0.34, -0.10]	-0.23 [-0.35, -0.10]
FEV1/FVC	-0.27 [-0.40, -0.12]	-0.25 [-0.38, -0.10]
FVC	0.00 [-0.17, 0.20]	0.01 [-0.17, 0.21]
height	-0.34 [-0.40, -0.26]	-0.34 [-0.40, -0.26]
smoking status	0.01 [-0.28, 0.40]	0.02 [-0.26, 0.40]
waist-hip ratio	-0.13 [-0.39, 0.22]	-0.12 [-0.38, 0.23]
allergic eczema	-0.66 [-0.87, -0.33]	-0.57 [-0.80, -0.24]
asthma	-0.07 [-0.45, 0.52]	-0.05 [-0.40, 0.48]
college education	-0.22 [-0.44, 0.06]	-0.20 [-0.41, 0.08]
hypertension	0.03 [-0.30, 0.48]	0.02 [-0.30, 0.46]
eosinophil count	-0.30 [-0.45, -0.12]	-0.28 [-0.43, -0.11]
high light scatter reticulocyte count	-0.42 [-0.55, -0.26]	-0.41 [-0.54, -0.25]
lymphocyte count	-0.40 [-0.52, -0.26]	-0.39 [-0.52, -0.25]
mean corpuscular hemoglobin	-0.35 [-0.45, -0.24]	-0.35 [-0.45, -0.23]
mean sphered cell volume	-0.34 [-0.47, -0.18]	-0.33 [-0.47, -0.17]
monocyte count	-0.14 [-0.29, 0.04]	-0.14 [-0.29, 0.04]
platelet count	-0.04 [-0.19, 0.13]	-0.04 [-0.19, 0.13]
platelet distribution width	-0.06 [-0.26, 0.17]	-0.05 [-0.23, 0.18]
red blood cell count	-0.30 [-0.43, -0.15]	-0.31 [-0.44, -0.16]
red blood cell distribution width	-0.15 [-0.31, 0.03]	-0.15 [-0.31, 0.04]
white blood cell count	-0.13 [-0.31, 0.10]	-0.11 [-0.29, 0.11]

α estimates calculated for 25 UK Biobank traits as shown in Table 2, only now without the use of LD weights. The second column shows results when using the default MAF cutoff of 0.07%, whereas the third column shows results for MAF $>$ 0.3%. Estimates are significantly higher when compared to estimates that include LD weights. The difference MAF cutoffs only have minimal impact on the estimates.

Supplementary Table 4: Heritability estimates for the 25 UK Biobank traits with and without LD and MAF correction

phenotype	h^2_{GCTA}	$h^2_{\alpha,\mathrm{noLD}}$	h^2_{α}
age of menarche	0.34 ± 0.02	0.23 ± 0.01	0.28 ± 0.01
blood pressure (diastolic)	0.32 ± 0.01	0.23 ± 0.00	0.27 ± 0.01
blood pressure (systolic)	0.31 ± 0.01	0.22 ± 0.00	0.26 ± 0.01
BMI	0.37 ± 0.01	0.27 ± 0.00	0.31 ± 0.00
bone mineral density	0.46 ± 0.01	0.34 ± 0.00	0.39 ± 0.00
FEV1/FVC	0.45 ± 0.01	0.31 ± 0.00	0.38 ± 0.01
FVC	0.42 ± 0.01	0.29 ± 0.00	0.33 ± 0.00
height	0.72 ± 0.01	0.55 ± 0.00	0.61 ± 0.00
smoking status	0.25 ± 0.03	0.25 ± 0.03	0.57 ± 0.02
waist-hip ratio	0.23 ± 0.01	0.16 ± 0.00	0.19 ± 0.00
allergic eczema	0.12 ± 0.01	0.09 ± 0.01	0.10 ± 0.01
asthma	0.12 ± 0.01	0.08 ± 0.00	0.10 ± 0.01
college education	0.15 ± 0.01	0.11 ± 0.00	0.15 ± 0.01
hypertension	0.21 ± 0.01	0.14 ± 0.00	0.16 ± 0.00
eosinophil count	0.31 ± 0.01	0.22 ± 0.00	0.25 ± 0.01
high light scatter reticulocyte count	0.34 ± 0.01	0.25 ± 0.01	0.29 ± 0.01
lymphocyte count	0.33 ± 0.01	0.25 ± 0.00	0.28 ± 0.01
mean corpuscular hemoglobin	0.45 ± 0.01	0.34 ± 0.00	0.35 ± 0.00
mean sphered cell volume	0.36 ± 0.01	0.26 ± 0.00	0.29 ± 0.01
monocyte count	0.33 ± 0.01	0.24 ± 0.00	0.25 ± 0.00
platelet count	0.44 ± 0.01	0.31 ± 0.00	0.35 ± 0.00
platelet distribution width	0.37 ± 0.01	0.25 ± 0.00	0.30 ± 0.00
red blood cell count	0.35 ± 0.01	0.26 ± 0.00	0.29 ± 0.01
red blood cell distribution width	0.36 ± 0.01	0.25 ± 0.00	0.26 ± 0.00
white blood cell count	0.33 ± 0.01	0.23 ± 0.00	0.25 ± 0.00

For each of the 25 UK Biobank traits the second column shows the SNP heritability estimate when using GCTA single variance component REML [1], the third column shows our MAF-corrected estimates, while the forth column shows our MAF and LD corrected estimates (see Online Methods). Heritability estimates for case-control traits are reported on the observed-scale [5]. In concordance to previous results [2] and our simulation results in Supplementary Table 2, correcting for MAF-dependent architectures leads to significantly lower heritability estimates. Combining MAF and LD correction leads to estimates that are slightly higher than only MAF corrected estimates. In cases where previous heritability estimates exist, our estimates are in good concordance [2;6]. Other methods of avoiding bias due to MAF-dependent and LD-dependent architectures have recently been proposed, including GREML-LDMS [2] and LDAK [3]; a complete benchmarking of SNP-heritability estimation methods will be provided elsewhere [4].

Supplementary Table 5: Joint estimation of the MAF-architecture parameter α and the LD-architecture parameter τ^* across 25 UK Biobank traits

phenotype	$\hat{\tau}^*$	$\Delta ll_{-0.15}$	$\Delta ll_{-0.3}$	$\Delta ll_{-0.45}$	$\Delta ll_{-0.6}$	$\hat{\alpha}$ at $\hat{\tau}^*$	s.e. of $\hat{\alpha}$
age of menarche	-0.30	8.92	9.29	-5.24	-30.08	-0.40	0.13
blood pressure (diastolic)	-0.30	25.01	30.98	-0.10	-56.59	-0.39	0.09
blood pressure (systolic)	-0.30	29.28	41.60	22.37	-18.26	-0.38	0.09
BMI	-0.15	24.69	16.10	–	-143.87	-0.17	0.08
bone mineral density	-0.30	70.82	105.47	70.27	-18.56	-0.35	0.06
FEV1/FVC	0.00	-26.97	-120.14	–	-406.83	-0.27	0.07
FVC	0.00	-29.18	-117.53	–	-398.63	0.00	0.10
height	-0.30	102.10	108.84	-60.10	-352.85	-0.45	0.03
smoking status	0.00	-3.53	-26.51	–	-111.77	0.01	0.17
waist-hip ratio	-0.30	16.60	18.01	-4.66	-40.10	-0.17	0.16
allergic eczema	-0.30	5.55	5.65	-3.26	-17.47	-0.61	0.15
asthma	0.00	-2.07	-11.00	–	-41.16	-0.07	0.25
college education	-0.15	14.87	12.66	–	-55.29	-0.26	0.12
hypertension	-0.30	11.89	14.49	0.35	-25.42	-0.18	0.18
eosinophil count	-0.15	18.37	6.27	–	-123.41	-0.35	0.08
high light scatter reticulociyte count	-0.15	15.95	1.58	–	-138.40	-0.48	0.07
lymphocyte count	-0.15	8.16	-20.32	–	-214.89	-0.46	0.07
mean corpuscular hemoglobin	-0.15	36.81	35.38	–	-161.52	-0.39	0.05
mean sphered cell volume	-0.30	34.38	42.68	4.91	-60.25	-0.43	0.07
monocyte count	-0.30	35.09	38.20	-12.74	-80.76	-0.19	0.09
platelet count	-0.30	56.35	75.66	28.41	-74.76	-0.19	0.08
platelet distribution width	-0.15	27.02	22.40	–	-114.21	-0.16	0.10
red blood cell count	-0.30	53.87	83.33	64.58	9.85	-0.39	0.07
red blood cell distribution width	-0.15	18.89	9.50	–	-136.51	-0.18	0.09
white blood cell count	0.00	-6.32	-55.34	–	-290.10	-0.13	0.10

We report joint estimates of α and τ^* for 25 UK Biobank traits. We tested (α, τ^*) pairs on a 2D grid, with $\tau^* = -0.60, -0.45, -0.30, -0.15, 0.00$ and $\alpha = -1.00, -0.95, -0.90, ...$ (the same set of α values as in the 1D analysis), reporting the τ^* value of the pair that maximizes the profile likelihood (see Online Methods). We also report estimates of $\hat{\alpha}$ at the best-fit τ^*, and their standard errors. We also report the difference in log likelihood (Δll) between each negative τ^* value and $\tau^* = 0$ at the respective $\hat{\alpha}$. We did not compute likelihoods at $\tau^* = -0.45$ for traits for which $\tau^* = -0.15$ attained a higher likelihood than -0.30 ($\Delta ll_{-0.45}$ indicated as –).

Supplementary Table 6: α estimates for 25 UK Biobank traits, only using common SNPs (MAF > 5%)

phenotype	$\hat{\alpha}$ [95% CI]	$\hat{\alpha}_{\text{common}}$
age of menarche	-0.40 [-0.63, -0.11]	-0.45 [-0.72, -0.11]
blood pressure (diastolic)	-0.39 [-0.54, -0.20]	-0.36 [-0.55, -0.13]
blood pressure (systolic)	-0.38 [-0.54, -0.18]	-0.36 [-0.56, -0.12]
BMI	-0.24 [-0.38, -0.06]	-0.19 [-0.37, 0.00]
bone mineral density	-0.35 [-0.45, -0.23]	-0.36 [-0.49, -0.23]
FEV1/FVC	-0.44 [-0.55, -0.31]	-0.43 [-0.57, -0.27]
FVC	-0.15 [-0.31, 0.04]	-0.11 [-0.29, 0.09]
height	-0.45 [-0.52, -0.39]	-0.43 [-0.51, -0.34]
smoking status	-0.16 [-0.43, 0.21]	-0.09 [-0.40, 0.30]
waist-hip ratio	-0.17 [-0.43, 0.19]	-0.07 [-0.37, 0.32]
allergic eczema	-0.60 [-0.85, -0.26]	-0.53 [-0.83, -0.13]
asthma	-0.25 [-0.60, 0.28]	-0.42 [-0.81, 0.12]
college education	-0.32 [-0.54, -0.04]	-0.29 [-0.52, -0.01]
hypertension	-0.18 [-0.46, 0.21]	-0.10 [-0.43, 0.33]
eosinophil count	-0.40 [-0.54, -0.24]	-0.42 [-0.58, -0.24]
high light scatter reticulocyte count	-0.53 [-0.65, -0.38]	-0.51 [-0.67, -0.33]
lymphocyte count	-0.52 [-0.63, -0.38]	-0.53 [-0.68, -0.36]
mean corpuscular hemoglobin	-0.42 [-0.53, -0.31]	-0.47 [-0.59, -0.34]
mean sphered cell volume	-0.43 [-0.56, -0.28]	-0.41 [-0.57, -0.23]
monocyte count	-0.19 [-0.35, -0.01]	-0.18 [-0.36, 0.02]
platelet count	-0.19 [-0.32, -0.03]	-0.14 [-0.29, 0.04]
platelet distribution width	-0.27 [-0.44, -0.07]	-0.12 [-0.32, 0.10]
red blood cell count	-0.39 [-0.51, -0.25]	-0.41 [-0.56, -0.24]
red blood cell distribution width	-0.20 [-0.36, -0.01]	-0.17 [-0.35, 0.03]
white blood cell count	-0.25 [-0.42, -0.03]	-0.16 [-0.36, 0.07]

α estimates were calculated for 25 UK Biobank traits with only SNPs of MAF > 5% used in the analysis.

Supplementary Table 7: $\hat{\alpha}$ and $\hat{\alpha}_{\text{common}}$ in simulated traits with threshold $T_{\text{sim}} = 0\%$, $T_{\text{sim}} = 5\%$, and $T_{\text{sim}} = 10\%$

	T_{sim}	τ_c^*	τ_{lf}^*	$\hat{\alpha}$	$\hat{\alpha}_{\text{common}}$	$\hat{\alpha}_{\text{common}} - \hat{\alpha}$	P-value
UK Biobank height	-	-	-	-0.45 [-0.52, -0.39]	-0.43 [-0.51, -0.34]	0.020 ± 0.052	-
simulations	0%	-0.3	-0.3	-0.45 [-0.51, -0.39]	-0.55 [-0.65, -0.45]	-0.103 ± 0.025	0.01
	5%	-0.3	-0.3	-0.38 [-0.44, -0.32]	-0.54 [-0.63, -0.45]	-0.164 ± 0.013	0.0002
	10%	-0.3	-0.3	-0.33 [-0.39, -0.27]	-0.52 [-0.60, -0.43]	-0.190 ± 0.020	0.0007
	0%	-0.4	0.0	-0.54 [-0.59, -0.48]	-0.55 [-0.63, -0.46]	-0.012 ± 0.068	0.7
	5%	-0.4	0.0	-0.44 [-0.50, -0.38]	-0.56 [-0.66, -0.47]	-0.128 ± 0.039	0.03
	10%	-0.4	0.0	-0.30 [-0.36, -0.23]	-0.44 [-0.54, -0.35]	-0.142 ± 0.051	0.04

We compared $\hat{\alpha}$ with $\hat{\alpha}_{\text{common}}$ (only using MAF $> 5\%$ SNPs during inference) in UK Biobank height to simulated traits mimicking UK Biobank height ($h^2 = 0.61$, $\alpha = -0.45$, sample size $N = 113,660$, and SNP number $M = 11,062,620$). For UK Biobank height, $\hat{\alpha}_{\text{common}} - \hat{\alpha}$ is 0.020 ± 0.052 (the standard error is conservative and based on s.e. for $\hat{\alpha}_{\text{common}}$ and $\hat{\alpha}$ respectively). We used $T_{\text{sim}} = 0\%$, $T_{\text{sim}} = 5\%$ and $T_{\text{sim}} = 10\%$ when simulating trait values (see Online Methods). We display mean $\hat{\alpha}$ and $\hat{\alpha}_{\text{common}}$ across 5 independent simulations each, with the boundaries of the 95% CI also being the average boundaries across simulations. Mean $\hat{\alpha}_{\text{common}} - \hat{\alpha}$ is reported with its standard deviation. In the last column we report a P-value indicating if $\hat{\alpha}_{\text{common}} - \hat{\alpha}$ is significantly different in the respective simulations compared to UK Biobank height. To do that we compare the difference between the respective mean $\hat{\alpha}_{\text{common}} - \hat{\alpha}$ values over the standard deviation across the 5 repetitions with an empirical null distribution assuming a normally distributed $\hat{\alpha}_{\text{common}} - \hat{\alpha}$. We also tested different LD-dependent architectures that are consistent with a genome-wide $\tau^* = -0.3$ (ref.[7]; also see Supplementary Table 5). In the main simulations $\tau^* = -0.3$ for all SNPs, in others τ^* differs between low-frequency SNPs (MAF $< 5\%$; τ^*_{lf}) and common SNPs (MAF $\geq 5\%$; τ^*_{c}). We chose $\tau^*_{\text{lf}} = 0.0$ and $\tau^*_{\text{c}} = -0.4$ since they correspond to a heritability weighted average τ^* of -0.3. We note that $|\tau^*_{\text{lf}}| < |\tau^*_{\text{c}}|$ is plausible since τ^* is defined as the effect of one standard deviation change in level of LD[7], which corresponds to a much larger LD score change in common SNPs than in rare SNPs. More extreme differences in LD-dependence are unlikely, since $\tau^* > 0$ is not consistent with the population genetic reasons for LD-dependent architecture[7]. Using $\tau^*_{\text{lf}} = 0.0$ and $\tau^*_{\text{c}} = -0.4$ and $T_{\text{sim}} = 0\%$ is consistent with the UK Biobank height result, however other changes to the model assumptions might also explain the discrepancy between UK Biobank height and the main simulations.

Supplementary Table 8: Estimates of τ for 25 UK Biobank traits

phenotype	$\hat{\alpha}$ [95% CI]	$\hat{\tau}$ [95% CI]
age of menarche	-0.40 [-0.63, -0.11]	0.44 [0.34, 0.52]
blood pressure (diastolic)	-0.39 [-0.54, -0.20]	0.43 [0.21, 0.67]
blood pressure (systolic)	-0.38 [-0.54, -0.18]	0.42 [0.19, 0.67]
BMI	-0.24 [-0.38, -0.06]	0.26 [0.10, 0.47]
bone mineral density	-0.35 [-0.45, -0.23]	0.38 [0.23, 0.54]
FEV1/FVC	-0.44 [-0.55, -0.31]	0.49 [0.31, 0.68]
FVC	-0.15 [-0.31, 0.04]	0.17 [0.00, 0.71]
height	-0.45 [-0.52, -0.39]	0.50 [0.36, 0.63]
smoking status	-0.16 [-0.43, 0.21]	0.23 [0.00, 0.50]
waist-hip ratio	-0.17 [-0.43, 0.19]	0.24 [0.00, 0.51]
allergic eczema	-0.60 [-0.85, -0.26]	0.68 [0.15, 1.00]
asthma	-0.25 [-0.60, 0.28]	0.35 [0.00, 0.77]
college education	-0.32 [-0.54, -0.04]	0.36 [0.10, 0.64]
hypertension	-0.18 [-0.46, 0.21]	0.26 [0.00, 0.56]
eosinophil count	-0.40 [-0.54, -0.24]	0.44 [0.24, 0.65]
high light scatter reticulocyte count	-0.53 [-0.65, -0.38]	0.61 [0.39, 0.83]
lymphocyte count	-0.52 [-0.63, -0.38]	0.59 [0.38, 0.80]
mean corpuscular hemoglobin	-0.42 [-0.53, -0.31]	0.47 [0.30, 0.63]
mean sphered cell volume	-0.43 [-0.56, -0.28]	0.48 [0.28, 0.68]
monocyte count	-0.19 [-0.35, -0.01]	0.21 [0.00, 0.45]
platelet count	-0.19 [-0.32, -0.03]	0.21 [0.00, 0.38]
platelet distribution width	-0.27 [-0.44, -0.07]	0.30 [0.10, 0.53]
red blood cell count	-0.39 [-0.51, -0.25]	0.43 [0.25, 0.61]
red blood cell distribution width	-0.20 [-0.36, -0.01]	0.22 [0.00, 0.45]
white blood cell count	-0.25 [-0.42, -0.03]	0.27 [0.00, 0.50]

Estimates of τ given inference of α for each of the 25 UK Biobank traits. Estimates of τ are calculated using results in Figure 3 and were constraint to [0,1]. Credible intervals were calculated using both the uncertainty in α and the uncertainty in the α-τ mapping (see Online Methods).

Supplementary Table 9: α estimates using the LDAK software

α	h_g^2	poly-genicity	imput. noise	LD dep. effects	mean $\hat{\alpha}_{\text{LDAK}}$
-0.3	0.4	1%	no	yes	0.139 ± 0.017
0	0.4	1%	no	yes	0.421 ± 0.018
-0.6	0.4	1%	no	yes	-0.184 ± 0.013
-0.3	0.2	1%	no	yes	0.130 ± 0.026
-0.3	0.4	100%	no	yes	0.070 ± 0.013
-0.3	0.4	1%	no	no	0.204 ± 0.017

The table shows mean α and heritability estimates using the LDAK software[3]. Phenotypes were simulated like in our main simulations analysis. Due to computational constraints we did not use the LDAK imputed genotype workflow, but instead used hard called genotypes for both phenotype simulation and estimation, an option provided by LDAK. Across these simulations, LDAK overestimates α by roughly 0.4. We note that ref.[3] suggest using $\hat{\alpha} = -0.25$ due to their cross-trait analysis, which is higher than our cross-trait estimate by only 0.13. However, this does not contradict our findings, since in their analysis $\hat{\alpha} = 0$ does not have a significantly lower likelihood.

Supplementary Table 10: Fixed effects accounted for in 25 UK Biobank traits

phenotype	fixed effects
age of menarche	10 PCs, assessment center
blood pressure (diastolic)	sex, 10 PCs, assessment center, age, BMI
blood pressure (systolic)	sex, 10 PCs, assessment center, age, BMI
BMI	sex, 10 PCs, assessment center, age
bone mineral density	sex, 10 PCs, assessment center, age, weight
FEV1/FVC	sex, 10 PCs, assessment center, age, height, smoking status
FVC	sex, 10 PCs, assessment center, age, height, smoking status
height	sex, 10 PCs, assessment center, age
smoking status	sex, 10 PCs, assessment center
waist-hip ratio	sex, 10 PCs, assessment center, age, BMI
allergic eczema	sex, 10 PCs, assessment center, age
asthma	sex, 10 PCs, assessment center, age
college education	sex, 10 PCs, assessment center, age
hypertension	sex, 10 PCs, assessment center, age
eosinophil count	sex, 10 PCs, assessment center, age
high light scatter reticulocyte count	sex, 10 PCs, assessment center, age
lymphocyte count	sex, 10 PCs, assessment center, age
mean corpuscular hemoglobin	sex, 10 PCs, assessment center, age
mean sphered cell volume	sex, 10 PCs, assessment center, age
monocyte count	sex, 10 PCs, assessment center, age
platelet count	sex, 10 PCs, assessment center, age
platelet distribution width	sex, 10 PCs, assessment center, age
red blood cell count	sex, 10 PCs, assessment center, age
red blood cell distribution width	sex, 10 PCs, assessment center, age
white blood cell count	sex, 10 PCs, assessment center, age

Fixed effects that were included in the REML analysis for each trait. Age, height, BMI and smoking status were included as the original and square value. Individuals taking blood pressure medications had 10mmHg (diastolic) or 15mmHg (systolic) added to their blood pressure trait value.

Supplementary References

[1] Jian Yang, S Hong Lee, Michael E Goddard, and Peter M Visscher. GCTA: a tool for genome-wide complex trait analysis. *The American Journal of Human Genetics*, 88:76–82, 2011.

[2] Jian Yang, Andrew Bakshi, Zhihong Zhu, Gibran Hemani, Anna AE Vinkhuyzen, Sang Hong Lee, Matthew R Robinson, John RB Perry, Ilja M Nolte, Jana V van Vliet-Ostaptchouk, et al. Genetic variance estimation with imputed variants finds negligible missing heritability for human height and body mass index. *Nature Genetics*, 47:1114–1120, 2015.

[3] Doug Speed, Na Cai, Michael R Johnson, Sergey Nejentsev, David J Balding, UCLEB Consortium, et al. Reevaluation of SNP heritability in complex human traits. *Nature Genetics*, 49:986–992, 2017.

[4] Luke M Evans, Rasool Tahmasbi, Scott I Vrieze, Gonçalo R Abecasis, Sayantan Das, Steven Gazal, Douglas W Bjelland, Teresa R Candia, Michael E Goddard, Benjamin M Neale, et al. Comparison of methods that use whole genome data to estimate the heritability and genetic architecture of complex traits. *Nature genetics*, 50(5):737, 2018.

[5] Sang Hong Lee, Naomi R Wray, Michael E Goddard, and Peter M Visscher. Estimating missing heritability for disease from genome-wide association studies. *The American Journal of Human Genetics*, 88:294–305, 2011.

[6] William J Astle, Heather Elding, Tao Jiang, Dave Allen, Dace Ruklisa, Alice L Mann, Daniel Mead, Heleen Bouman, Fernando Riveros-Mckay, Myrto A Kostadima, et al. The allelic landscape of human blood cell trait variation and links to common complex disease. *Cell*, 167:1415–1429, 2016.

[7] Steven Gazal, Hilary K Finucane, Nicholas A Furlotte, Po-Ru Loh, Pier Francesco Palamara, Xuanyao Liu, Armin Schoech, Brendan Bulik-Sullivan, Benjamin M Neale, Alexander Gusev, et al. Linkage disequilibrium–dependent architecture of human complex traits shows action of negative selection. *Nature genetics*, 49(10):1421, 2017.

[8] Benjamin C Haller and Philipp W Messer. SLiM 2: Flexible, interactive forward genetic simulations. *Molecular Biology and Evolution*, 34:230–240, 2016.

[9] Adam R. Boyko, Scott H. Williamson, Amit R. Indap, Jeremiah D. Degenhardt, Ryan D. Hernandez, Kirk E. Lohmueller, Mark D. Adams, Steffen Schmidt, John J. Sninsky, Shamil R. Sunyaev, Thomas J. White, Rasmus Nielsen, Andrew G. Clark, and Carlos D. Bustamante. Assessing the evolutionary impact of amino acid mutations in the human genome. *PLOS Genetics*, 4:1–13, 05 2008.
